# Probing the opto-electronic, phonon spectrum, and thermoelectric properties of lead-free fluoride perovskites A_2_GeSnF_6_ (A = K, Rb, Cs) for energy harvesting devices

**DOI:** 10.1038/s41598-024-61210-3

**Published:** 2024-06-02

**Authors:** Danish Abdullah, Dinesh C. Gupta

**Affiliations:** Condensed Matter Theory Group, School of Studies in Physics, Jiwaji University, Gwalior, 474011 India

**Keywords:** Phonon spectrum, Non-magnetic semiconductors, Direct bandgap, Optoelectronic device applications, Thermoelectric properties, Materials science, Physics

## Abstract

The present work employs density functional theory to explore the structural, optoelectronic, and thermoelectric attributes of the halide-based double perovskite A_2_GeSnF_6_ (A = K, Rb, and Cs) compounds. The stable phonon dispersion spectrum affirms dynamical stability, whereas the enthalpy of formation and tolerance factor evaluated collectively verify structural stability. Considering the Tran Blaha modified Becke Johnson potentials (mBJ), the predicted direct band gaps along the symmetry point are 3.19 eV for K_2_GeSnF_6_, 3.16 eV for Rb_2_GeSnF_6_ and 3.12 eV Cs_2_GeSnF_6_. According to an in-depth examination of the optoelectronic features, A_2_GeSnF_6_ (A = K, Rb, and Cs), double perovskites are assuring contenders for optoelectronic devices due to their suitable bandgap. The extremely high figure of merit values (0.94–0.97) obtained from the numerical calculation of power factor and thermal conductivity suggest the intriguing prospects of these compositions for thermoelectric devices. These studies offer a perceptive comprehension of the materials for their potential applications in the future.

## Introduction

Double perovskite materials have been immensely probed in recent years owing to their diverse geometries and diversified composition. These compounds’ magnificent capabilities, inherited from their diverse structural makeup, ensure that they are ideal for photoelectronic, microelectronic, and photovoltaic (PV) applications^1,2^. Among double perovskites, halide-based double perovskites acquired enormous popularity. According to reports, these halide perovskites could replace lead perovskites. These compounds have been examined due to their remarkable characteristics, which made them desirable for photovoltaic and optoelectronic applications. Despite having the most significant recorded efficiency^1^ for solar cells, lead toxicity and low stability^3–6^ are both of the main issues hindering lead halide perovskites from being tremendously commercialized. The lead concentrations and the duration of exposure could culminate in symptoms that encompass damage to neurons to fatalities^7^. To tackle the rising demand for green energy resources, one crucial possibility is to glance for lead-free and stable replacements that possess similar optical and thermoelectric properties as lead-containing halide perovskites^8–10^. Lead-free double perovskites with the formula A_2_BB^**′**^X_6_ could be considered^11–14^ due to their numerous uses in environmentally friendly and renewable energy. A is a cation from the s/p block, B and B^**′**^ are cations from the p/d/f block, and X is an anion (oxide or halide ion) in A_2_BB^**′**^X_6_^15–17^. The enduring popularity of this type of compound can be attributed to its superior physical characteristics, which consist of suitable and tunable band gaps, low effective charge carrier masses, high optical absorption coefficients, long diffusion lengths, high defect tolerance, and compositional flexibility^5,17–19^.

Since the double perovskites are quaternary compounds, countless combinations may be adopted, each with distinct attributes of optoelectronic and thermoelectric properties. These materials have been rigorously investigated in recent years for photoluminescence, photovoltaic, and thermoelectric applications^11,12,16,20–26^. The double perovskites comprising Ge have been illustrated to be extraordinary materials. Following the literature review, Cs_2_GeSnX_6_ (X = Cl, Br, I) has a high absorption coefficient as well as substantial reflectivity, resulting in an excellent material for optoelectronic applications including solar and photovoltaic cells. Furthermore, given that the determined figures of merit (zT) for Cs_2_GeSnCl_6_, Cs_2_GeSnBr_6_, and Cs_2_GeSnI_6_ are 0.97, 0.96, and 0.89, respectively, these materials exhibit potential for thermoelectric applications^27^. At 1000 K, the most significant computed values of zT are 0.417 and 0.395 for Rb_2_GeSnCl_6_ and Rb_2_GeSnBr_6_, respectively. These values are significantly greater than those perceived in traditional perovskite halides^28,29^. As a result, both Rb_2_GeSnX_6_^30^ double perovskites appear promising for thermoelectric power generation. In addition, the highest values of ε1(ω) for the assessed perovskites are within the visual range of electromagnetic radiations. This guarantees that these materials have an optimal visible spectrum response. The lead-free halide double perovskites K_2_GeSnBr_6_ and K_2_GeSnI_6_^31^ have a significant coefficient of absorption and reflectivity, implying that they are optimal for optoelectronic applications such as solar and photovoltaic cells. Moreover, X_2_GeSnI_6_ (X = Rb, Cs)^32^ was explored by Malik Azmat Ali et al., in which he predicted the negative formation energies and positive phonon frequencies to indicate their structural and dynamic stabilities. The electronic structure calculation shows direct bandgaps of 0.49 eV for Rb_2_GeSnI_6_ and 0.57 eV for Cs_2_GeSnI_6_, suggesting semiconducting behavior and its potential use in photovoltaics. The examined thermoelectric properties of both compounds imply their potential use in thermoelectric devices. The analysis provided above emphasizes the potential advantages of utilizing Ge-based double perovskites as an alternative to lead-based perovskites. A_2_GeSnF_6_(A = K, Rb, Cs) are yet unexplored members of this group. It is vital to investigate these double perovskites for low-cost green and renewable energy production. Consequently, an in-depth inquiry into these compounds’ structural, electronic, and optical properties using density functional theory (DFT) has been accomplished. Boltzmann’s transport theory offers precise predictions about thermoelectric characteristics. This investigation addresses the theoretical gap in the body of expertise and will serve as a framework for further research on A_2_GeSnF_6_(A = K, Rb, Cs) perovskites compounds. Our findings demonstrate the viability of A_2_GeSnF_6_(A = K, Rb, Cs) compounds for use in thermoelectric, solar, and electronic devices.

## Theoretical approach to studying computational aspects

The simulations have been carried out within the DFT framework adopting the FP-LAPW formulation in WIEN2K^33^. The PBE-GGA exchange-correlation potential has been employed to optimize the cubic structure and volume^34^. To compensate for GGA-PBE’s inadequacies, the band gap and optical characteristics have been computed by deploying the mBJ exchange-correlation potential^35^. A linearized set of plane-wave basis has been utilized to determine the electron behavior inside the muffin-tin sphere and interstitial space. The expansion of these base sets was regulated by retaining R_MT_ Kmax at 7 and l_max_ at 10, where K_max_ is the most significant value of k and R_MT_ is the smallest muffin-tin radius. The minimal energy was chosen at 6.0 Ry for the acceptable energy convergence criterion, and 1000 k-points were chosen. The energy and charge convergence prerequisites were established at 0.0001 Ry and 0.0001e, respectively. Semi-classical Boltzmann transport theory with constant proximity, as deployed in BoltzTraP2^36^, has been applied to compute the thermoelectric traits. Employing the improved unit cells, we applied self-consistent simulations to generate ground state wave functions with a more constrained convergence threshold of 10^–18^ for potential residual. This case study explored the appropriate implications of density functional perturbation theory (DFPT), as outlined in Quantum Espresso, in assessing the dynamical stability within the primitive unit cells of K_2_GeSnF_6_, Rb_2_GeSnF_6_ and Cs_2_GeSnF_6_ perovskites. The mBJ potential was implemented for this scrutiny, and self-consistent perturbation operations were performed with a convergence threshold of 10^–18^ for the potential residual to generate the dynamic matrix.

### Structural properties

The volume optimization approach via PBE-GGA^33^ potential was executed to compute the structural parameters of A_2_GeSnF_6_(A = K, Rb, Cs). Table 1 shows the computed a_0_ (lattice parameter), V_0_ (volume), E_0_ (ground-state energy), B (bulk modulus), and B′** (**Pressure derivative of Bulk-modulus). For efficient use in devices, thermodynamic stability must be satisfied. If the synthesis reaction is exothermic, the material is deemed stable. The enthalpy of formation (ΔH) can be exploited to determine the type of reaction. Exothermic reactions have a negative enthalpy of formation. The following equation was implemented to compute the enthalpy of the formation of A_2_GeSnF_6_(A = K, Rb, Cs).

1 $$\Delta {\text{E}} = {\text{E}}_{{{\text{Total}}}} - {\text{aE}}_{{\text{A}}} - {\text{ bE}}_{{\text{B}}} - {\text{ cE}}_{{{\text{B}}^{\prime } }} - {\text{ dE}}_{{\text{X}}}$$

E_Total_ is the total energy of A_2_GeSnF_6_ (A = K, Rb, Cs), where E_A_, E_B_, E_B′_, and E_F_ possess the energy of A = (K, Rb, Cs), Ge, Sn, and F, respectively. Energy values extracted for A_2_GeSnF_6_ (A = K, Rb, Cs) are − 2.07, − 1.61, and − 1.14, respectively. The fact that the enthalpy of production is negative ensures that A_2_GeSnF_6_(A = K, Rb, Cs) is thermodynamically stable. K_2_GeSnF_6_ is the most stable in comparison, followed by Rb_2_GeSnF_6_ and Cs_2_GeSnF_6_. The following equation was implemented to compute the Goldschmidt tolerance (τ_F_) and the octahedral factor (μ) to acquire the cubic phase stability of A_2_GeSnF_6_(A = K, Rb, Cs).

2 $$\uptau _{F} = { }\frac{{\left( {{\text{r}}_{{\text{A}}} { } + {\text{ r}}_{{\text{F}}} } \right){ }}}{{ \sqrt {2{ }\left( {\frac{rGe + rSn}{2}{ } + {\text{ rF}}} \right)} }}{ }\,{\text{and }}\,{ }\mu = \frac{{R_{Ge} + R_{Sn} }}{{2R_{F} }}$$

**Table 1:** The determined values of lattice constant (a_0_), volume(V), Bulk-modulus (B), Pressure derivative of Bulk-modulus (B′), ground state energy E_0_, Enthalpy(eV), tolerance factor (τ_F_), octahedral factor (μ).

Compounds	a_0_(Å)	V (a.u^3^)	B (GPa)	B′	E_0_ (Nm)	ΔH	τ_F_	Echo (eV)	μ
K_2_GeSnF_6_	10.21	1793.26	37.98	5.15	− 76,304.46	− 2.07	1.06	2.79	0.53
Rb_2_GeSnF_6_	10.75	2093.54	32.50	5.21	− 102,046.02	− 1.61	1.04	2.24	0.53
Cs_2_GeSnF_6_	11.49	2561.22	26.64	5.37	− 156,194.81	− 1.14	1.02	1.54	0.53

The atomic radii of the A (K, Rb, Cs) and F atoms are represented by r_A_ and r_F_, respectively, and the average atomic radius of the Ge and Sn atoms is represented by r_B`_.

**Figure 1:**
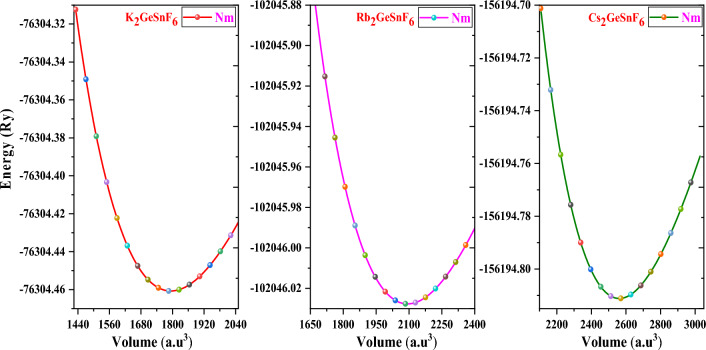
Optimization plot of investigated double perovskites A_2_GeSnF_6_ (A = K, Rb, Cs).

**Figure 2:**
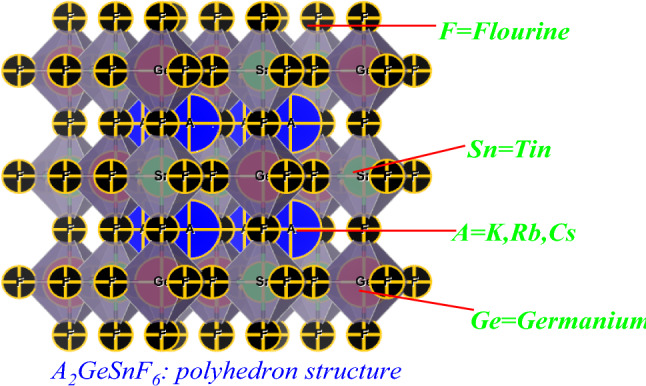
polyhedron structure of the compound A_2_GeSnF_6_ (A = K, Rb, Cs).

The values of τ_F_ and μ should, respectively, stay between the ranges of 0.81–1.1 and 0.44–0.90 for a stable cubic phase^37^. Table 1 lists the computed Goldschmidt tolerance (τ_F_) and octahedral factor (μ) for A_2_GeSnF_6_(A = K, Rb, Cs). According to our calculations, A_2_GeSnF_6_(A=K, Rb, Cs) is stable in the cubic phase. The optimization plot and structure of A_2_GeSnF_6_ are depicted in Figs. 1 and 2.

Cohesive energy (Echo) is the degree of energy required to disintegrate a solid into its atoms or critical structural units. This energy serves as vital for compound stability. The cohesive energy of a molecule is enhanced as it gains stability. This energy gauges the degree of atomic bonding in the material. For double perovskites, the cohesive energy can be estimated as

3 $$E_{coh}^{{A2BB^{\prime } X6}} = \frac{{2E_{atom}^{A} + E_{atom}^{B} + E_{atom}^{{B^{\prime } }} + 6E_{atom}^{X} - E_{{A2BB^{\prime } X6}} }}{10}$$

Table 1 discloses the assessed value of the presented double perovskite A_2_GeSnF_6_(A = K, Rb, Cs). The outcomes suggest that the atoms interact strongly bonded to create the crystal.

### Phonon stability

**Figure 3:**
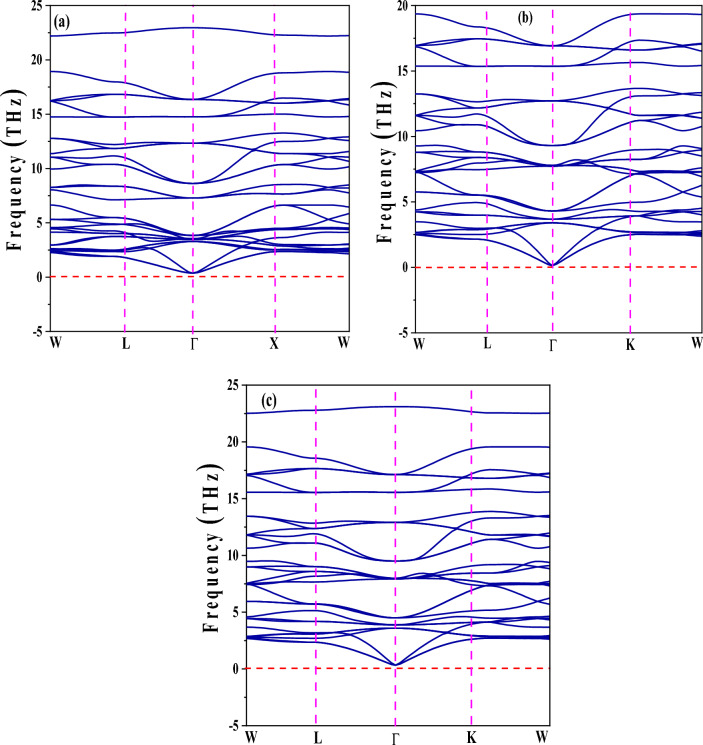
Phonon spectrum Calculations For double perovskites of A_2_GeSnF_6_: (**a**) K_2_GeSnF_6_, (**b**): Rb_2_GeSnF_6_, (**c**): Cs_2_GeSnF_6_.

Phonons possess critical roles in the dynamics of structural stability, thermal properties, and structural stability, all of which are vital components in fundamental materials science challenges. In quantum physics, a phonon is the intrinsic vibrational motion that happens whenever a lattice of atoms or molecules vibrates continuously at a single frequency. The exploration of whether a crystal lattice is dynamically stable, that is, whether the lattice vibrations or phonons in the crystal are stable and well-defined, is referred to as phonon stability. A stable and dynamic crystal lattice specifies that the phonon frequencies are realistic (non-complex) and positive, signifying that atoms steadily oscillate around their equilibrium positions. One may determine the phonon dispersion and look at the phonon frequencies across the Brillouin zone to ascertain phonon stability. The crystal lattice is dynamically stable if every frequency is positive and real. On the other hand, the existence of imaginary frequencies suggests instability and the requirement for more investigation. The non-existence of imaginary frequencies in Fig. 3 ensures phonon stability and validates the dynamical stability of the system. The phonon stability of A_2_GeSnF_6_(A = K, Rb, Cs) turns out by evidence of real frequencies.

### Mechanical stability

The behavior of a material under various external constraints (loads, forces, pressures) is determined by elastic parameters. These parameters elucidate the material’s resistance against the external load and the limits within which the materials are mechanically functional. Mechanical stability is one of the necessities that materials must possess so that they can be used in engineering construction and smart material technology. Elastic constants (EC) act as pathways as they are related to various thermodynamic parameters which in turn are related to the material’s physical properties. The mechanical stability is determined by obtaining elastic stiffness coefficients Cij’s. These Cijs are highly dependent on the symmetry of the crystal structure. The number of Cijs for cubic crystals is three that is C_11_, C_12,_ and C_44_ Where C_11_ represents the longitudinal compression or elongation i.e., alteration along the uniaxial direction, and describes the hardness of the material. C_12_ represents the transverse expansion (distortion) and is related to Poisson’s ratio. C_44_ defines the shear elastic parameter as it depends on the shear modulus. Thus, one has to be very careful while estimating these parameters using DFT methods because we need more precise methods to evaluate the total energy or stress accompanying strain. The values of ECs for the alloys under investigation are presented in Table 2. From where it is evident that all are positive and follow the Born-Haung criteria; *C*_44_ > 0; *C*_11_ + 2*C*_12_ > 0; C_11_ − *C*_12_ > 0^38^. From these parameters, we can elucidate other elastic parameters. Formalism namely shear and bulk modulus are determined by implementing the Viogt-Reuss-Hill averaging scheme. For cubic crystals, Voigt constraints are;

4 $$B_{V} = \frac{{\left( { C_{11} + 2C_{12} } \right)}}{3}$$

5 $$G_{V} = \frac{{\left( { C_{11} - C_{12} + 3C_{44} } \right)}}{5},\,{\text{ respectively}}.$$

**Table 2:** Calculated values of elastic constants C_11_, C_12_, C_44_ in (GPa), Young’s modulus Y (GPa), bulk modulus B (GPa), shear modulus G (GPa), B/G ratio, Poisson’s ratio (ν) and Cauchy pressure (C_P_) of compound A_2_GeSnF_6_(A = K, Rb, Cs).

Compounds	C_11_	C_12_	C_44_	Y	B	G_V_	G_R_	B/G	ѵ	Cp
K_2_GeSnF_6_	93.18	25.63	7.81	40.11	48.14	11.27	18.19	3.26	0.36	17.82
Rb_2_GeSnF_6_	85.81	28.07	10.70	43.46	47.31	14.30	17.96	2.93	0.34	17.37
Cs_2_GeSnF_6_	81.58	31.47	13.87	47.10	48.17	16.88	18.34	2.93	0.33	17.60

The Reuss formalism is given by;

6 $$B_{V} = B_{R} ;\,G_{R} = \frac{{5\left( { C_{11} - C_{12} } \right) C_{44} }}{{4C_{44} + 3\left( {C_{11} - C_{12} } \right) }}$$

while the Hill’s approximation is obtained by averaging both Voigt and Reuss methods^39,40^;

7 $$B = \user2{ }\frac{{B_{V} + B_{R} }}{2}$$

8 $$G = \user2{ }\frac{{G_{V} + G_{R} }}{2}$$

The shear and bulk moduli represent the rigidity and compressibility of the alloys. The obtained values of both parameters are presented in Table 2. These values suggest that alloy resists the shape and volume deformations and is also compressible as the values of B are adequate.

Some other important elastic parameters, such as Poisson’s ratio (ʋ) and Young’s modulus (*Y)* can be calculated by using the relations.

9 $$Y = \frac{9BG}{{3B + G}}$$

10 $$\upupsilon = \frac{3B - 2G}{{2\left( {3B + G} \right)}}$$

Young’s modulus (Y) defines the stiffness of the materials and is defined as the ratio of stress to strain. Large values of Y affirm the brittle nature of materials while small value embodies ductile character. This further can be authenticated by Cauchy’s pressure and Pugh’s ratio. The malleability, ductility, or brittleness can be characterized by calculating Pugh’s ratio (B/G, limiting value 1.75) and Cauchy’s pressure (C_P_ = C_12_–C_44_)^41,42^. The values obtained are presented in Table 2, it is evident that B/G is greater than 1.75 for both alloys, and also C_P_ is positive indicating the ductile nature of the alloys.

## Results and discussions

### Electronic properties

**Figure 4:**
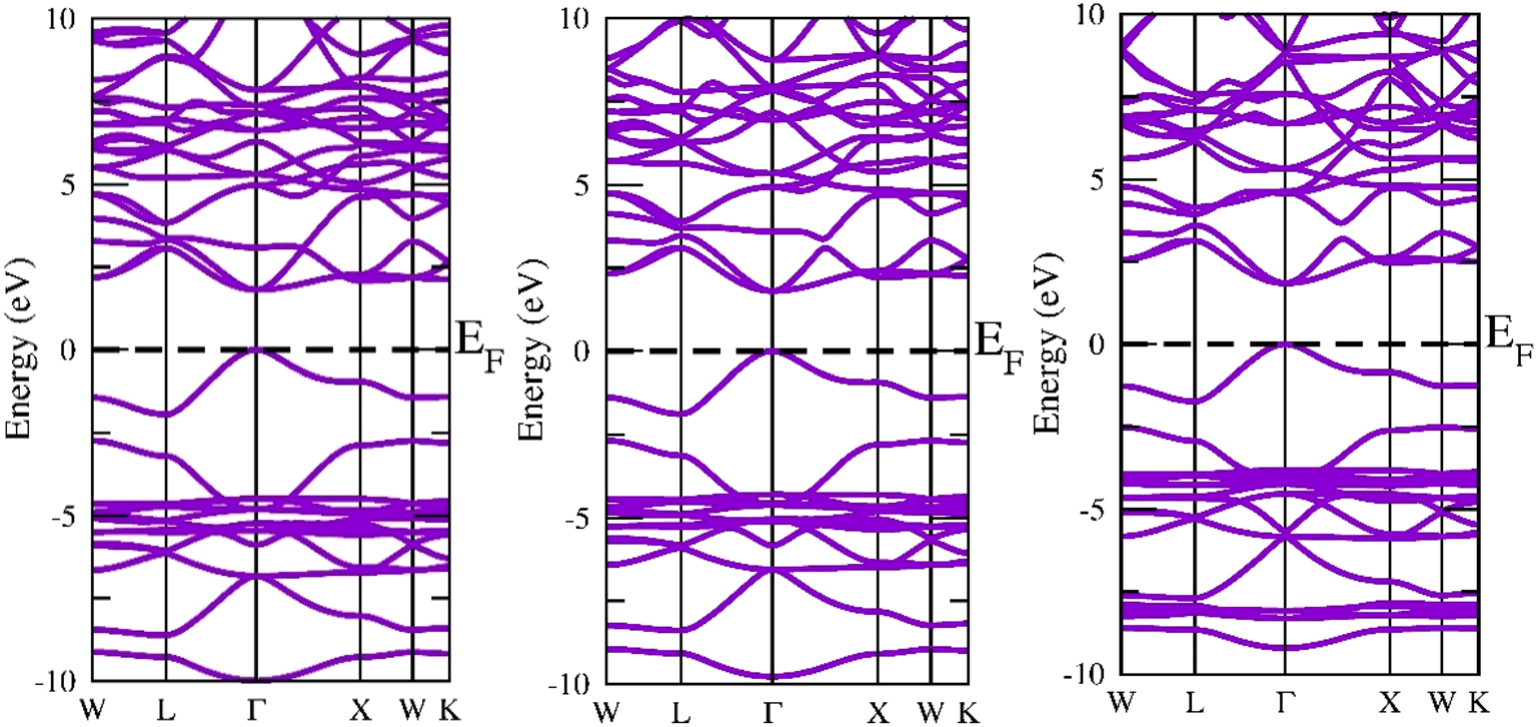
GGA band approximation of A_2_GeSnF_6_(A = K, Rb, Cs).

**Figure 5:**
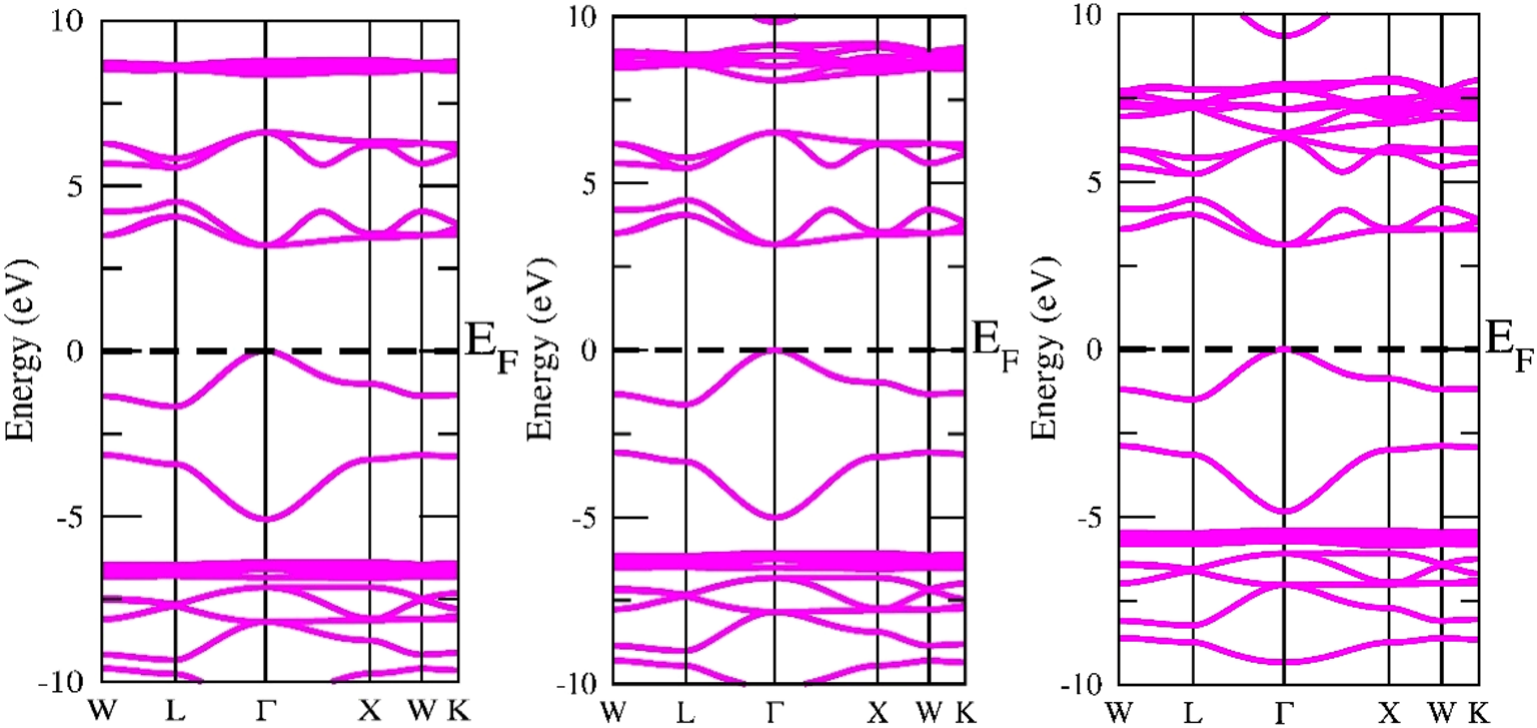
GGA + mBJ band approximation of A_2_GeSnF_6_(A = K, Rb, Cs).

**Figure 6:**
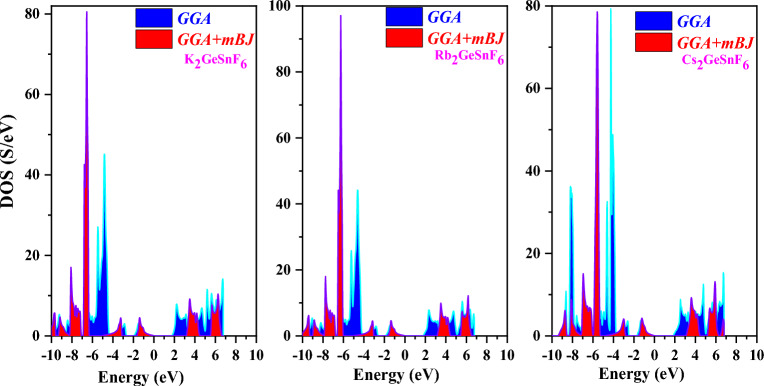
Total density of states of compound A_2_GeSnF_6_(A = K, Rb, Cs) using mBJ approach.

**Figure 7:**
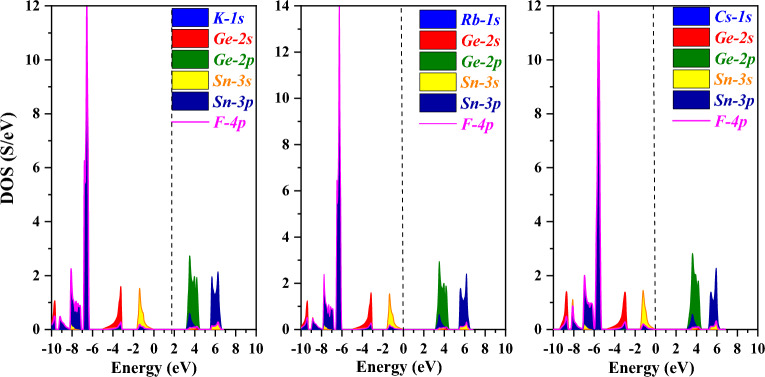
partial density of states of compound A_2_GeSnF_6_(A = K, Rb, Cs) using mBJ approach.

The band structure is crucial due to the fact it may convey a material’s intrinsic physical characteristics, such as its thermoelectric, optical, photocatalytic, and other traits^43^. In the present research, GGA and mBJ have been employed to recognize the band structures of A_2_GeSnF_6_(A = K, Rb, Cs) while taking into account the optimized unit cell. The predicted electronic states for GGA (violet) and MBJ (Magenta) are displayed in Figs. 4 and 5. A_2_GeSnF_6_(A = K, Rb, Cs) have valance band maxima and conduction minima that occur at the same symmetry point. For solar cell applications, direct band gap materials are believed to be the most suitable^44^. As a result, the researched materials may be exploited for manufacturing solar cells. To comprehend the band structure, the total and partial density of states (DOS) have been determined and provided in Figs. 6 and 7. This governs the impact of various states in the valence and conduction bands. Figure 6 reveals that the lower conduction band is dominated by A-d states, with modest contributions from p and d levels of Ge and Sn atoms, whereas the upper conduction band is dominated by p states of Ge, with a fairly tiny contribution from Sn-p states. For these double perovskites, the halide atom delivers the most significant impact in the middle of the valence band. The s states of the Sn atom supreme in the valence band maximum. As a consequence, alterations and recombination are likely between the Sn-s and Ge/Sn-p orbitals, which could point out optical and thermoelectric properties.

### Optical properties

Optical properties were rigorously studied to understand the crucial nature of the projected material for solar cell applications. The optical performance of the transition study from the valence to the conduction band was investigated. The relationship between light and materials shows optical properties. Optoelectronic devices’ light absorption and emission intensities rely on intraband and interband transition^45^. The complex dielectric (CD) function ε(ω), absorption coefficient α(ω), complex refractive index n(ω), and reflectivity R(ω) have all been researched in the context of optoelectronic characteristics.

**Figure 8:**
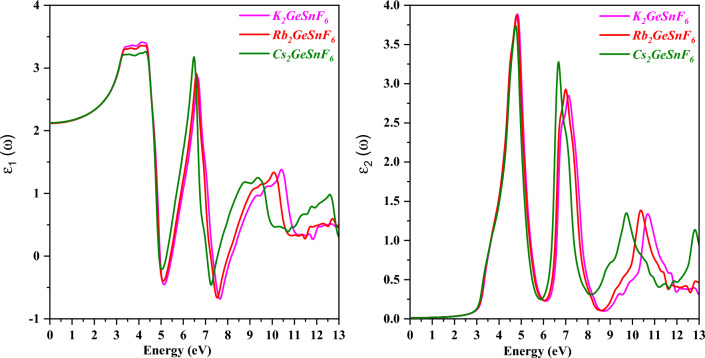
Variation of optical properties with a real (ε1(ω)) and imaginary (ε2(ω)) dielectric coefficient of double perovskites A_2_GeSnF_6_(A = K, Rb, Cs) compounds, respectively.

The dielectric function’s real and imaginary components are denoted by ε1 and ε2 and can be expressed as ε(ω) = ε1(ω) + iε2(ω) and determined for A_2_GeSnF_6_(A = K, Rb, Cs) compounds. The spectra appear in Fig. 8. A material’s electronic BS (band structure) is directly linked to the real component of the dielectric function, which serves to specify the extent to which it can be polarized. The dielectric function’s real portion also clarifies its dispersive behavior. On the other hand, the imaginary part exhibits the way the material absorbs light. Figure 7 depicts the optical activity of the compounds from 0 to 13 eV, with multiple prominent peaks^46^.

**Figure 9:**
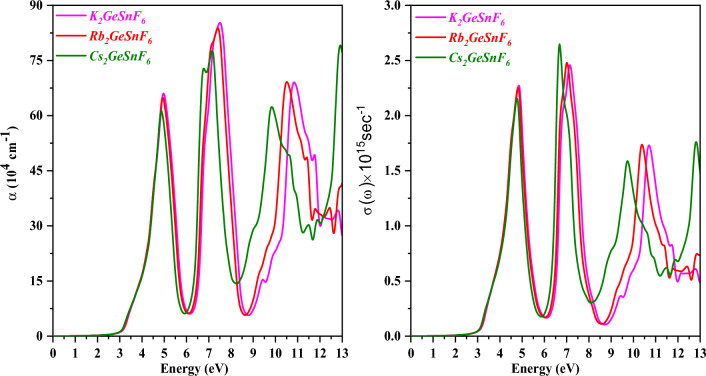
Variation of absorption coefficient (α(ω)) and optical conductivity (σ(ω)) with a photon energy of compounds A_2_GeSnF_6_(A = K, Rb, Cs) respectively.

The absorption coefficient α(ω), which is another metric in addition to the dielectric tensor, is vital in deciding how a material will behave in optoelectronic applications. α(ω)^47,48^ reveals insight into how deeply a specific wavelength traverses a substance before being absorbed. Figure 9 displays the A_2_GeSnF_6_(A = K, Rb, Cs) absorption spectrum. Significant intensities appear in the 0 to 13 eV energy range of the absorption spectra. The maximum solar energy intensity is believed to remain within the energy range. A vital gauge of charge carriers’ movement to optical frequencies is optical conductivity σ(ω). Figure 9 demonstrates the simulated plots of σ(ω) for A_2_GeSnF_6_(A = K, Rb, Cs) double perovskites. Values of σ(ω) are zero below the optical band gap, as revealed by Fig. 8. This shows that there needs to be more excitement among charge carriers to participate in electrical transport. The charge carriers are excited by the optical photon, which has energy equal to the optical band gap, and σ(ω) receives its values. when photons of an appropriate frequency incident on a material surface, σ(ω) likewise follows an identical pattern to that of ε2(ω)^49^ due to the simultaneous occurrence of absorption, transmission, reflection, and conduction.

**Figure 10:**
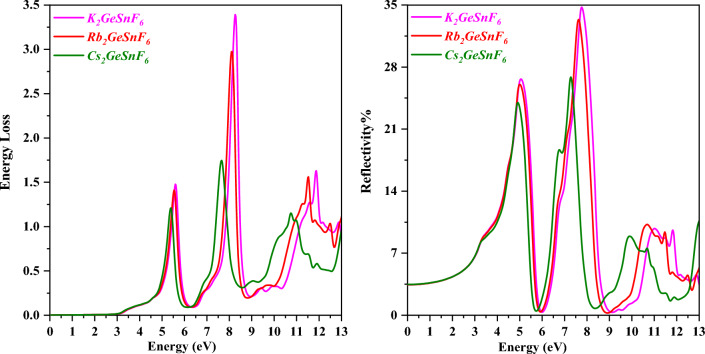
Variation of Energy Loss and Reflectivity with a photon energy of compounds A_2_GeSnF_6_(A = K, Rb, Cs) respectively.

Reflectivity R(ω) is a gauge of how well photons reflect off a substance’s surface. It is an amount of electromagnetic energy that is reflected from a compound’s surface^50^. The anticipated R(ω) is displayed in Fig. 10. The static reflectivity values (R (0)) for A_2_GeSnF_6_(A = K, Rb, Cs) have been predicted to be 5%, employing this figure. R (0) exhibits the same pattern as ε1(0) in this case. The observable range of the R(ω) contains values below 35%. This suggests that the explored double perovskites, A_2_GeSnF_6_(A = K, Rb, Cs), can absorb the maximum number of visible photons. So, we endorse these materials for application in solar cell applications. The energy loss function evaluates the energy lost by a fast-moving electron as it traverses through a material. The energy loss function for the oxide complexes A_2_GeSnF_6_(A = K, Rb, Cs) is displayed in Fig. 10. There is barely any energy loss in the area where absorption is most significant. In the specified energy range, several peaks have been noticed. K_2_GeSnF_6_, Rb_2_GeSnF_6,_ and Cs_2_GeSnF_6_’s highest peak values can be detected at 8.47 eV, 8.10, and 7.55 eV, respectively.

**Figure 11:**
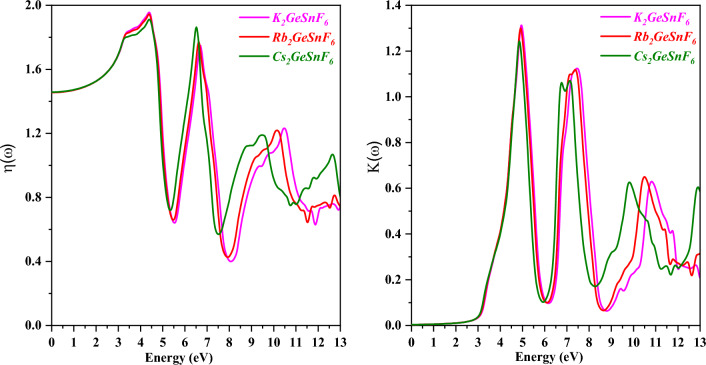
Variation of Refractive index n(ω) and Extinction coefficient K(ω) with a photon energy of compounds A_2_GeSnF_6_(A = K, Rb, Cs) respectively.

The n(ω) (refractive index) is an essential term to comprehend when determining the level of refraction considering it is so advantageous for photoelectric applications. The refractive index of a substance examines how light interacts with it. Light penetrates more gradually in materials with high refractive indices, which results in a proportionately higher alteration in the direction of the light interior of the material. The estimated refractive indexes for the main compounds are shown in Fig. 11. At zero energy, K_2_GeSnF_6_, Rb_2_GeSnF_6_, and Cs_2_GeSnF_6_ have static reflection coefficients of 1.438, 1.439, and 1.440, respectively. K_2_GeSnF_6_ has a maximum refractive index of 1.95 at 4.48 eV, Rb_2_GeSnF_6_ has a maximum refractive index of 1.96 at 4.49 eV, and Cs_2_GeSnF_6_ has a maximum refractive index of 1.90 at 4.5 eV. The refractive index of a material dictates how much light penetrates when passing through it. The lighter is refracted the higher the coefficient. Any strategy that improves the electron density in a substance frequently triggers the refractive index to rise. The extinction coefficient is a parameter that indicates how well a material absorbs or reflects radiations or light at a certain wavelength. The results of calculating the extinction coefficient K(ω) for compounds A_2_GeSnF_6_(A = K, Rb, Cs) are shown in Fig. 11. K_2_GeSnF_6_ has a local maximum extinction coefficient of around 1.32 at 5.0 eV, while Rb_2_GeSnF_6_’s is approximately 1.30 at 4.99 eV, and for Cs_2_GeSnF_6_ is 1.25 at 4.97 eV.

### Thermoelectric properties

By implementing thermoelectric materials, surplus heat can be diverted into advantageous electrical energy. Perovskites are more desirable for it owing to their affordability, low price, intense electrical conductivity, and friendly to the environment^51^. As a consequence, the BoltzTraP code incorporates the Wien2k output files of structural and electronic data as input files for computing the thermoelectric parameters for A_2_GeSnF_6_(A = K, Rb, Cs) double perovskites, involving electronic and lattice thermal conductivities, electrical conductivity, carrier per unit volume, Seebeck coefficient, and dimensionless figure of merit^52^. The TE properties of A2GeSnF6(A = K, Rb, Cs) double perovskites are examined to assess the functionality with which thermal energy may be converted into electrical power. Waste heat energy can be incorporated into electricity utilizing TE materials to assist in tackling the energy problem and reduce pollution^53,54^.

**Figure 12:**
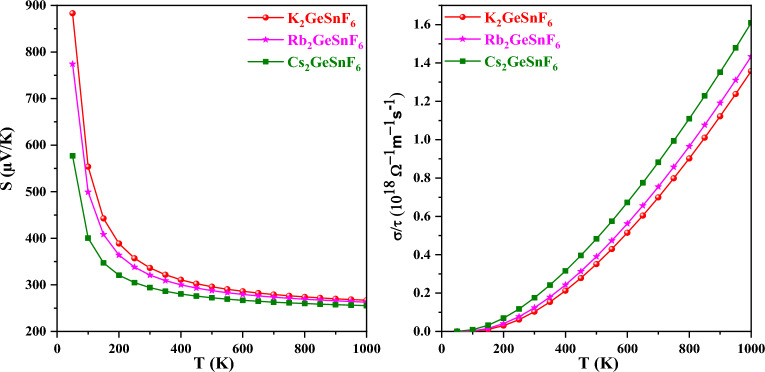
Variation of electrical conductivity and see beck coefficient with temperature for A_2_GeSnF_6_(A = K, Rb, Cs).

Figure 12 discloses the computed electrical conductivity. An increase in temperature is spurred on by the carriers migrating from the VB to the CB more effortlessly as the temperature rises. The rising conductivity validates these compounds’ semiconducting properties. Due to the increase in atomic size, which gives Coulomb repulsion to the electrons and restricts the carrier’s motion, σ/τ reduced as Cs was swapped out for Rb and K, respectively. Equation σ = *ne*μ states that charge carrier concentration improves with temperature, increasing electrical conductivity. The variables σ, n, e, and μ stand for electrical conductivity, charge carrier concentration, an electron’s charge, and mobility, respectively. The rise in electrical conductivity with temperature clarifies the negative temperature coefficient of resistance that reflects the semiconducting characteristic of these materials A2GeSnF6(A = K, Rb, Cs). For A_2_GeSnF_6_(A = K, Rb, Cs), the high electrical conductivity also implies a low electrical resistivity (ρ = 1/σ). An upsurge in temperature triggers more charge carriers between the valence and conduction bands in semiconductors. This increases the values of electrical conductivity (σ). This behavior is clarified by an increase in thermal energy, that raises the mobility of charge carriers in the material. Rising thermal vibrations interrupt the crystal lattice when the temperature grows, enabling less scattering and enhanced charge carrier mobility. As a consequence, the electrical conductivity spikes. The calculated values are listed in the Table 3.

**Table 3:** Computed values of Electrical conductivity(σ/τ), Seebeck coefficient(S), electronic thermal conductivity (κ_e_/τ), and figure of merit (zT).

Compounds	Electrical conductivity At 1000 K (Ω^−1^ m^−1^ s^−1^)	Seebeck coefficient At 50 K (μVK^−1^)	Electronic thermal conductivity At 1000 K (WK^−1^ m^−1^ s^−1^)	zT	
				50 K	1000 K
K_2_GeSnF_6_	1.36 × 10^18^	883	1.21 × 10^14^	0.97	0.79
Rb_2_GeSnF_6_	1.43 × 10^18^	774	1.25 × 10^14^	0.96	0.78
Cs_2_GeSnF_6_	1.61 × 10	577	1.34 × 10^14^	0.94	0.77

The Seebeck effect, identified by German scientist Thomas Joan Seebeck in 1821, is utilized for operating thermoelectric generators^53^. The Seebeck coefficient gauges the potential difference between the two distinct semiconductors/conductors^54^ when a temperature gradient is introduced between two junctions. When a temperature gradient is brought about to a material, charge carriers mobilized by higher temperatures in the warmer region traverse to the colder region, enhancing electron concentration and leading to the Seebeck effect. Due to the two distinguished interactions of metal temperatures, the potential gradient influences the thermoelectric efficiency of the material and is thus measured by employing the Seebeck coefficient (S)^54^. The electronic motion that generates the voltage (in μVK^−1^) yields the thermo-electromotive force. The generated voltage is determined by the composition of the material and the electrical mobility within the material. An excellent thermoelectric device dictates a high S value because it relies on the zT. The Seebeck coefficient and electrical conductivity, as measured by the power factor PF = S^2^σ^55^, characterize the thermoelectric efficiency of semiconductors. At 50 K, the Seebeck coefficient of the A_2_GeSnF_6_(A = K, Rb, Cs) perovskite is S = 883, 774, and 577 μVK^−1^. After approaching 250 K, it begins to diminish, eventually becoming roughly constant and attaining values of 274, 269, and 256 μVK^−1^ at 1000 K. Due to the inverse relationship between the Seebeck coefficient and charge carriers, the Seebeck coefficient (S) showed a diminishing trajectory as temperature grew within the 50–1000 K temperature range. Because the electrical conductivity increases as the charge carrier rises, the Seebeck coefficient must drop as the temperature rises, as shown in Fig. 12, confirming its semiconductor nature. The Seebeck coefficient readings reflect a reduction with increasing temperature. Increased temperature leads to an immense rise in carrier density, culminating in a decreasing Seebeck coefficient (S) value.

**Figure 13:**
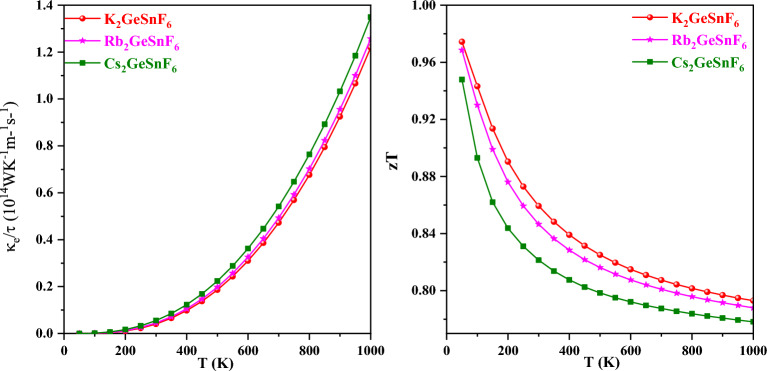
Variation of electronic thermal conductivity and figure of merit zT with temperature for A_2_GeSnF_6_(A = K, Rb, Cs).

The assessed electronic thermal conductivity κ_e_ is displayed in Fig. 13. This parameter estimates how much charge carriers assist in heat transmission. To put it another way, it quantifies how efficiently a material transmits heat. Low κ_e_ values must be attained for perfect thermoelectric efficiency. As can be observed from Fig. 13, these compounds’ κe values rise as temperature rises. This is a typical trait of semiconductors that has also been identified in numerous other types of materials^56,57^.

zT, generally referred to as the figure of merit, gauges a thermoelectric material’s aptitude for conversion^58,59^. The compound achieves the greatest efficiency when zT is equal to or larger than unity. The values of each thermoelectric parameter for A_2_GeSnF_6_(A = K, Rb, Cs) are displayed in Table 3. Based on the published results, the compounds under scrutiny may be deemed the most suitable for implementation in thermoelectric and optoelectronic devices.

### Lattice thermal conductivity and power factor

**Figure 14:**
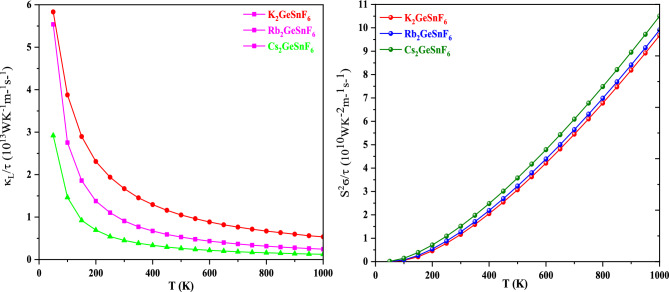
Variation of Lattice thermal conductivity(κ_L_/τ) and power factor(S^2^σ/τ) with temperature For A_2_GeSnF_6_(A = K, Rb, Cs).

As Boltzmann’s equation calculates only the electronic part, Slack’s equation^60^ is used to determine the lattice part

11 $$K_{L} = \frac{{A\theta_{D}^{3} V^{1/3} M}}{{\gamma^{2} N^{2/3} T}}$$

where A is a constant of value 3.0 × 10^–8^, M is molar mass, V is volume, γ is the Grüneisen parameter, and N is the number of atoms in a unit cell. since lattice thermal conductivity is inversely proportional to temperature; that is, with an increase in temperature, lattice thermal conductivity decreases (see Fig. 14). Moreover, we have also calculated the power factor as revealed in Fig. 14. The thermoelectric efficiency can be gauged by computing the power factor (PF = S^2^ σ/τ) that composes the numerator of the figure of merit expression. Figure 14 depicts that PF increases, as the temperature increases from 300 to 1000 K, respectively. The increasing PF with increasing temperature indicates that investigated double perovskites remain attractive at elevated temperatures due to their potential for wasted heat to electricity conversion applications. The evaluated values are listed in Table 4.

**Table 4:** Computed values of power factor(S^2^σ/τ) and lattice thermal conductivity (κ_L_/τ) temperature For A_2_GeSnF_6_(A = K, Rb, Cs).

Compounds	Lattice thermal conductivity (WK^−1^ m^−1^ s^−1^)		Power factor (WK^−2^ m^−1^ s^−1^)	
	50 K	1000 K	300 K	1000 K
K_2_GeSnF_6_	5.53 × 10^13^	0.24 × 10^13^	1.16 × 10^10^	9.67 × 10^10^
Rb_2_GeSnF_6_	5.83 × 10^13^	0.53 × 10^13^	1.27 × 10^10^	9.95 × 10^10^
Cs_2_GeSnF_6_	2.91 × 10^13^	0.12 × 10^13^	1.51 × 10^10^	10.50 × 10^10^

## Conclusion

This study examined the optical, electronic, and thermoelectric properties of the double perovskite A_2_GeSnF_6_(A = K, Rb, Cs) compound using the mBJ potential. The tolerance factor (1.02–1.06) confirms the structure’s stability, whereas the negative sign (−) of enthalpy of formation energy demonstrates thermodynamic reliability and the phonon spectrum supports dynamical stability. The direct bandgaps for the energy band structures were 3.19 eV (K_2_GeSnF_6_), 3.16 eV (Rb_2_GeSnF_6_), and 3.12 eV (Cs_2_GeSnF_6_). The electronic band structures clarify the compound’s semiconducting features. The value of high absorption coefficient, high electrical conductivity, and low value of reflectivity suggest its potential uses in solar cell technology. Also, the value of the band gap for these materials occurs in the range of the visible spectrum reflecting its potent use in photovoltaic gadgets. zT values for K_2_GeSnF_6_, Rb_2_GeSnF_6_, and Cs_2_GeSnF_6_ are reported as 0.97, 0.96, and 0.94, respectively. The present predictions will be essential in future experimental and theoretical research of perovskite materials for consumption in electrical device applications.

## Data Availability

The datasets generated and/or scrutinized during the current study would be available from the corresponding author upon reasonable request.
